# Music training and empathy positively impact adults’ sensitivity to infant distress

**DOI:** 10.3389/fpsyg.2014.01440

**Published:** 2014-12-19

**Authors:** Christine E. Parsons, Katherine S. Young, Else-Marie E. Jegindø, Peter Vuust, Alan Stein, Morten L. Kringelbach

**Affiliations:** ^1^Department of Psychiatry, University of Oxford, Oxford, UK; ^2^Centre of Functionally Integrative Neuroscience, Aarhus University, Aarhus, Denmark; ^3^Department of Psychology, University of California at Los Angeles, Los Angeles, CA, USA; ^4^The Royal Academy of Music, Aarhus/Aalborg, Denmark

**Keywords:** parenting, crying, vocalization, music, empathy, emotion perception, parent-infant, vocal emotion perception

## Abstract

Crying is the most powerful auditory signal of infant need. Adults’ ability to perceive and respond to crying is important for infant survival and in the provision of care. This study investigated a number of listener variables that might impact on adults’ perception of infant cry distress, namely parental status, musical training, and empathy. Sensitivity to infant distress was tested using a previously validated task, which experimentally manipulated distress by varying the pitch of infant cries. This task required that participants discriminate between pitch differences and interpret these as differences in infant distress. Parents with musical training showed a significant advantage on this task when compared with parents without. The extent of the advantage was correlated with the amount of self-reported musical training. For non-parents, individual differences in empathy were associated with task performance, with higher empathy scores corresponding to greater sensitivity to infant distress. We suggest that sensitivity to infant distress can be impacted by a number of listener variables, and may be amenable to training.

## INTRODUCTION

Crying is a powerful communicator of infant need. Parents’ capacity to perceive and respond to infant communication is an important determinant of the quality of early care (Parsons et al., 2010). This is important because there is good evidence that early caregiving shapes infant development across a range of domains including cognitive, social, and emotional functioning (Raviv et al., 2004; Sohr-Preston and Scaramella, 2006; Stein et al., 2008). Of all of the typical infant behaviors, crying presents a substantial challenge to parents. Hearing the sound is associated with a cascade of neuroendocrinal events, most notably autonomic arousal and brainstem activity (Parsons et al., 2012, 2013c) Crying can cause parents substantial distress, especially when perceived as excessive or difficult to sooth (Miller et al., 1993) and is associated with parental sense of competence (e.g., Stifter et al., 2003).

The capacity to respond appropriately to infant crying depends on basic perceptual abilities. A number of acoustic parameters within infant cries have been identified that provide information as to the infant’s current physiological and affective state. These include pitch, duration, onset, and pauses (for review, see Soltis, 2004). Of these, pitch has been identified as the key acoustic factor affecting caregiver perceptions and responses (LaGasse et al., 2005). Evidence for the role of pitch as a marker of infant distress comes from studies examining adults’ perception of infant emotion in natural contexts (e.g., Zeskind and Collins, 1987), studies that experimentally manipulate pitch and measure adults’ responses (e.g., Zeskind et al., 1995) and studies of infant pain responses (e.g., Porges, 1995).

### PITCH AND INFANT DISTRESS

While much of the evidence for pitch as a marker of infant distress comes from studies manipulating cry acoustics, there is some work examining naturally occurring crying. For instance, in one study of caregiving behavior in a daycare setting, adults were found to respond with greater urgency and make additional efforts to soothe high pitched crying when compared to lower pitched crying (Zeskind and Collins, 1987). Another study found that the cries of high medical complication infants were characterized by a higher pitch compared with low complication infants and rated as more urgent, aversive, grating, sick, distressing, piercing, and ‘discomforting’ by adult listeners (Zeskind and Lester, 1978). Finally, one study examined a number of acoustic parameters of infant crying and found that pitch was the best predictor of adults’ perception of distress (Zeskind and Marshall, 1988).

A body of laboratory studies have provided clear evidence that experimental manipulations of pitch result in cries being perceived as increasingly arousing, urgent, aversive, and sick sounding as pitch increases (Zeskind et al., 1995; Dessureau et al., 1998). Some studies manipulated the infant cry pitch by specific frequency amounts (e.g., 100 Hz; Zeskind et al., 1995), or specific semitone amounts (Young et al., 2012) or percentage of overall pitch (Protopapas and Eimas, 1997), but all have yielded consistent results.

There are clear physiological mechanisms linking pitch, and changes in pitch, to infant affective state (Soltis, 2004). The association between pitch and distress is mediated by the vagus, in particular the branch of the vagus linking the nucleus ambiguous to the larynx. During acute stress (e.g., pain or fear), the sympathetic nervous system is activated and the parasympathetic nervous system is attenuated. As part of the parasympathetic withdrawal, vagal tone is lowered, eliciting a number of physiological adaptations (Porter et al., 1988; Porges, 1995, 1997). Among these adaptations, muscle tension is crucial, resulting in changes in the pitch of vocalizations. Greater infant arousal and distress is associated with increased tension in the vocal chords, producing higher pitched vocalizations (Porter et al., 1988; Porges, 1995).

Observational studies of infant pain response and arousal provide further confirmation of this physiological link. For instance, cry pitch has been shown to directly relate to parasympathetic activity in the child (Porter et al., 1988; Green et al., 2000). In addition, pitch is higher when infants cry in response to painful medical procedures (injection) compared to non-painful procedures (alcohol rub; Grunau et al., 1990). Finally, cries during invasive circumcisions are higher in pitch than cries during less invasive circumcisions (Porter et al., 1986). These converging lines of evidence underline the significance of pitch as a marker of infant distress. It would seem important, therefore, that adults be able to perceive and respond to cry pitch. Recent work has attempted to identify factors that might negatively impact infant cry perception generally, and also specifically in relation to pitch. There is some evidence for the effects of illegal drug use (Schuetze and Zeskind, 2003), teenage motherhood (Giardino et al., 2008), and depression, with depression being the most studied factor. Several studies have reported that mothers with depression are less likely to initiate appropriate caregiving responses to their infant’s cries than healthy mothers (Bettes, 1988; Murray et al., 1993; Schuetze and Zeskind, 2001). While there are a number of plausible features of depression that might impact on a mother’s response to her infant, it has been suggested that mothers with depression might have decreased sensitivity to basic perceptual features of these sounds, particularly pitch (Zeskind et al., 1995; Donovan et al., 1998) which may impair initiation of responding. This suggestion is supported by work showing decreased ability to discriminate distress, as manipulated by pitch, in adults with depression and no music training (Young et al., 2012). Perceptual difficulties, which may be linked to other core features of depression such as motivation and biased information processing, might be important in mother-infant interactions in depression.

### PARENTAL STATUS AND RESPONDING TO CRYING

There has been a general assumption that becoming a parent might positively impact adults’ responses to infant cries. Studies to date have yielded somewhat divergent results. For instance, there is some evidence that parents rate infant crying as less distressed (Irwin, 2003) and less aversive than non-parents (Zeskind and Lester, 1978), and respond with less cardiac reactivity (Out et al., 2010). It has also been reported that mothers tend to respond with greater sympathy and alertness to infant cries compared to non-mothers (Stallings et al., 2001).

However, other studies have reported no difference between mothers and non-mothers on measures of mean heart rate in response to infant crying (Hall and Morsbach, 1989), and that non-parents are more similar in cardiac response to experienced parents than new parents (Boukydis and Burgess, 1982). Another study reported that parents and non-parents did not differ in their ratings of infant distress, empathic concern, caregiving intention (Lin and McFatter, 2012) or motor movements in response to infant cries (Parsons et al., 2012). Furthermore, brain imaging studies have not yet provided a clear picture (Parsons et al., 2013a,b). One fMRI study reported stronger activation to infant crying in the amygdala and limbic areas in parents compared to non-parents (Seifritz et al., 2003), but another recent study reported no such difference (De Pisapia et al., 2013).

### EMPATHY AND CAREGIVING

The reasons for these discrepant findings are unclear, but may be related to individual differences in the participant groups included in these studies. For instance, individual differences in adults’ own early life experiences have also been shown to moderate responses to infant cues (Bhandari et al., 2014a,b). Furthermore, it has been argued that individual differences in dispositional empathy might moderate caregiving responses to distressed others (Lockwood et al., 2013). However, studies of the effects of parenthood have not yet addressed individual differences in empathy directly. This seems important because recent models of empathy have emphasized its role in motivating parental caregiving behavior (Decety and Cowell, 2014). There is substantial overlap in the neurochemical underpinnings of caregiving behavior and empathic responding. Oxytocin, in particular, has been strongly linked to both caregiving (Rilling and Young, 2014; Bakermans-Kranenburg and van Ijzendoorn, 2008; Gordon et al., 2010; Feldman et al., 2013; but effects are not necessarily straightforward, Voorthuis et al., 2014) and empathic responding in humans (Rodrigues et al., 2009; Hurlemann et al., 2010).

### MUSICAL TRAINING: AN ADVANTAGE IN EMOTION PROCESSING?

Another plausible factor that might impact on the perception of infant crying is musical experience. Emotion perception in vocal sounds and music depend on shared acoustic and neural mechanisms (Nair, 2002; Scherer, 2003). Numerous studies have shown that musicians have heightened sensitivity to emotion in speech compared to non-musicians (for review, see Kraus and Chandrasekaran, 2010) and emerging evidence suggests that this sensitivity extends to non-speech vocalizations also (Strait et al., 2009; Young et al., 2012). Indeed, pitch processing is one acoustic component where musicians show clear advantages over non-musicians (Magne et al., 2006; Musacchia et al., 2007; Vuust et al., 2012), which may be especially helpful for responding to infant crying.

In the current study, we aimed to examine the effects of parental status, empathy, and musical training on the perception of distress in infant cries. While previous work has tended to examine factors such as parental status in isolation, we tested whether individual differences in dispositional empathy and years of musical training might also be important. While one previous study demonstrated the importance of musical training in adults experiencing current psychopathology (Young et al., 2012), we tested whether it might be of relevance in healthy adults. Using a two-alternative forced choice (2AFC) task, we asked participants to identify the more distressed of two infant cries. The two sounds differed only with respect to their pitch, which was systematically altered by varying amounts (as described in Young et al., 2012). We expected that (i) parents would show greater sensitivity to infant distress compared with non-parents, (ii) musicians would show greater sensitivity than non-musicians, and (iii) dispositional empathy would correlate with sensitivity.

## MATERIALS AND METHODS

Ethical approval for the study was granted by the Ethics Committee of Central Region Denmark.

### PARTICIPANTS

Participants were recruited from the general community in Aarhus using posters, online advertisements, and social media. All provided written informed consent for participation. Inclusion criteria for participation were: not currently experiencing any psychological or physical conditions, no problems with hearing, normal vision or vision correct to normal. Fifty-seven women and 52 men participated, of whom 29 were mothers and 25 were fathers. All of the fathers and mothers had infants aged less than 18 months (*M* = 8.1 months, SD = 4.43). A total of 109 men and women participated (see Table 1 for demographic information). Participants were aged between 21 and 39 years (*M* = 28.76, SD = 3.69). For assessing music training, participants were asked “Have you received formal education in music (excluding primary school)?” Music training was defined as individuals who reported receiving 4 or more years of formal music training (Young et al., 2012). Adults who reported having less than this were recorded as having “no music.”

**Table 1:** Participant demographic information by parental status and music training.

	**Parents (*N* = 54)**		**Non-parent (*N* = 55)**	
	**Music (*N* = 25)**	**No music (*N* = 29)**	**Music (*N* = 26)**	**No music (*N* = 29)**
Mean years of music (SD)	6.80 (4.46)		6.6 (4.62)	
Mean BDI score (SD)	4.48 (3.8)	3.28 (2.86)	2.92 (2.94)	2.76 (2.57)
Mean EQ score (SD)	49.96 (8.67)	49.10 (12.11)	44 (10.38)	50.48 (8.29)

The Beck Depression Inventory-Second Edition (BDI-II) was used to screen for current depressive symptoms. The Empathy Quotient was used as a measure of self-reported dispositional empathy.

### QUESTIONNAIRES

Measures of current depressive symptoms and empathy were obtained using Beck Depression Inventory-Second Edition (BDI-II) and the Empathy Quotient (EQ). The BDI-II is a 21-item questionnaire, with each item scored on a 4-point scale indicating the presence and severity of symptoms (Beck et al., 1996). It is one of the most widely used measures of depression symptoms. Recent reviews indicate that it has high internal consistency and good test-retest reliability (consistency, 0.9, retest reliability ranges from 0.73 to 0.96; Wang and Gorenstein, 2013).

The EQ is also a widely used questionnaire (Baron-Cohen and Wheelwright, 2004), comprising 60 items (40 empathy related, 20 fillers), each scored on a 4-point scale. Each item linked to empathy is then associated with a score (0, 1, or 2), and a total score is computed (0–80). Three 5-item subscales (0–10) have been identified, cognitive empathy, emotional reactivity and social skills (Muncer and Ling, 2006). The EQ has high internal consistency (Cronbach’s alphas > 0.88 in both cases) and low levels of skew and kurtosis (Wakabayashi et al., 2006). The EQ questionnaire also shows moderate associations with the Interpersonal Reactivity Index (IRI; Davis, 1980), suggesting concurrent validity (Lawrence et al., 2004). EQ scores have also been shown to correlate with performance on tasks measuring non-verbal mental inference (the “Eyes” task; Lawrence et al., 2004), accuracy perceiving face gender (Penton-Voak et al., 2007) and face identify (Bate et al., 2010).

### STIMULI

Fifteen digital recordings of infant cry bursts from the OxVoc database (Parsons et al., 2014b) were used in this study. Audio recordings in this database were collected from nine healthy infants (age *M* = 6.7 months, SD = 0.9) filmed in their own homes during a play and feeding session with a caregiver. Individual cry clips were digitally altered to increase and decrease overall pitch by 0.25, 0.5, 1, and 2 semitones, using Adobe Audition software (CS5.5 v4.0; same procedure as in Young et al., 2012). This corresponded to changes in fundamental frequency of approximately 5–10, 12–17, 25–30, and 50–60 Hz, respectively. Clips of the same cry burst at different pitches were presented in pairs that differed by 0.5 semitones (–0.25 vs. +0.25), 1 semitone (–0.5 vs. +0.5), 2 semitones (–1 vs. +1), and 4 semitones (–2 vs. +2).

### PROCEDURE

In the 2AFC task, participants listened to two clips successively and were asked to choose which cry burst “sounded more distressed.” Each pitch difference (0.5, 1, 2, or 4 semitones) was presented for each of the 15 vocalizations, resulting in a total of 60 trials. Sounds were presented using Presentation software (Version 14.4, Neurobehavioral Systems, Inc., www.neurobs.com; 24-bit Realtek High Definition Audio sound card) through Sony in-ear earphones (MDR-EX77LP) using a desktop computer. To facilitate ease of responding, sounds were presented alongside an image of an unfamiliar fractal (a fragmented geometric shape) in one of two locations. Accuracy was defined as the number of trials in which participants identified the higher-pitched clip as “more distressed.” The order of whether the correct clip was presented first or second was randomized.

## RESULTS

**FIGURE 1:**
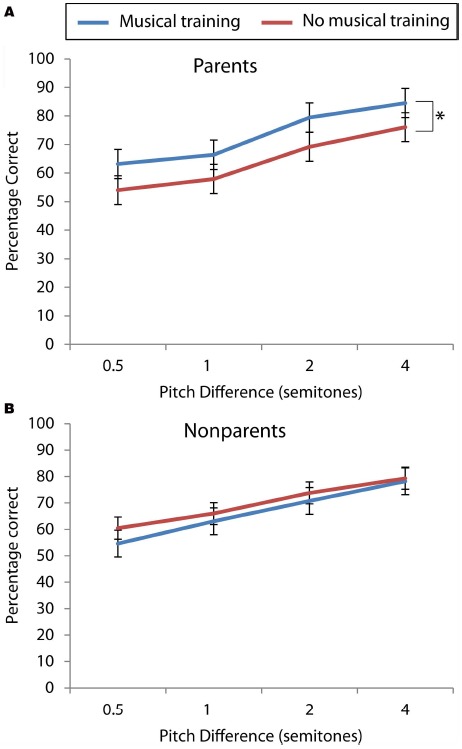
**Percentage of correct trials at each level of pitch difference for (A) parents and (B) non-parents, comparing adults with and without music training.** The scores of parents with music training were significantly higher than the scores of parents without music training, but for non-parents, there was no difference. * significant difference.

Figure 1 presents the percentage of correct trials at the four levels of pitch difference for the parent and non-parent groups separately, comparing those with and without music training. There were no significant differences between men and women overall (*p*-values >0.42). Therefore, results for the men and women are presented together.

A mixed ANOVA, with pitch difference as the within-subjects factor, parental status and music training as the between-subjects factors was used to examine participant accuracy scores. The variance in accuracy scores across the groups was found to be similar at all four difficulty levels of the task using Levene’s test (*p*-values >0.16). There was a significant effect of pitch level [*F*(3,193) = 54.68, *p* < 0.0001], with scores being highest on the largest pitch difference (2 semitones) and lowest for the smallest difference (0.25 semitone). There was a significant interaction between parental status and music training [*F*(1,104) = 9.92, *p* < 0.002], where parents with music training performed better than those without, but non-parents with and without music training showed no difference. None of the other main effects or interaction effects reached significance (all *p*-values >0.14).

### PARENTS WITH AND WITHOUT MUSIC TRAINING

Taking a total accuracy score (four levels summed, scores out of 60), parents with music training performed better (*M* = 44.04, SD = 7.28) than parents without [*M* = 38.3, SD = 5.63; *t*(52) = 3.22, *p* < 0.002]. The difference between the two groups was significant at all four levels of the task (see Figure 1A). Furthermore, in parents the number of years of music training positively correlated with overall scores (*r* = 0.24, *p* < 0.04) and scores at two levels of the task (2 semitone difference: *r* = 0.24, *p* < 0.04; 4 semitone difference: *r* = 0.26, *p* < 0.03). However, this was not the case for non-parents.

### EMPATHY

Table 2 presents correlations between participants’ EQ scores and their performance on the distress detection task. Taking participants’ total scores, EQ scores were positively correlated with task performance, *r* = 0.18, *p* = 0.029 (one-tailed test). None of the individual EQ subscales (Cognitive Empathy, Emotional Reactivity, Social Skills) were significantly correlated with accuracy on the task. Comparing correlations by parental status, the association between EQ scores and task accuracy appeared to be primarily driven by the non-parent group. For non-parents only, EQ was positively correlated with scores on the task overall, and at each level of the task (see Table 2). Examining the subscales of the EQ, Cognitive Empathy showed the strongest association with overall task performance (*r* = 0.355, *p* < 0.004), but the other two subscales were also significantly correlated with performance (Emotional Reactivity, *r* = 0.3, *p* < 0.01; Social Skills, *r* = 0.25, *p* < 0.03). For the parent group, EQ scores, or its subscales, did not correlate significantly with task performance.

**Table 2:** Results of correlation analyses of EQ scores with accuracy of performance on distress detection task.

	**Whole group**		**Parent**		**Non-parent**	
**Semitone difference**	***r***	***p***	***r***	***p***	***r***	***p***
0.5	0.1	0.16	0.02	0.43	0.24*	0.04
1	0.09	0.19	0.08	0.27	0.29*	0.02
2	0.15	0.07	0.02	0.43	0.32**	0.01
4	0.1	0.16	0.06	0.33	0.25*	0.03

*Correlation is significant at the 0.05 level (one-tailed).

**Correlation is significant at the 0.01 level (one-tailed).

## DISCUSSION

This study examined factors that might impact perception of emotion in infant vocal cues. The listener variables examined were parental status, music training, and self-reported empathy. The task required that adults discriminate differences in pitch and interpret these as differences in infant distress. There were two main findings. First, among parents, those with music training showed greater sensitivity to distress in infant cries, as manipulated by pitch, than those without music training. Second, for adults who were not parents, empathy was positively associated with sensitivity to distress. Music training and empathy appeared to only have measurable effects in the parent and non-parent groups, respectively.

### MUSIC TRAINING

For the parent group, even a relatively short duration of music training appeared to confer advantages in discriminating distress in infant cries. Music-trained participant groups in other studies often consist of professionals (e.g., Wallentin et al., 2010), those with a training onset early in life (e.g., Dohn et al., 2012) and/or a duration of longer than ten years (e.g., Schön et al., 2004; Thompson et al., 2004; Musacchia et al., 2007; Parbery-Clark et al., 2009). Adults categorized in this study as having music training had just 6–7 years on average, and there were no requirements as to its intensity, frequency, or onset. This is of interest because another study using approximately the same cutoff (6.5 years) did not find an advantage for those with music training on an emotional prosody task (Trimmer and Cuddy, 2008). It may be that for some tasks, a more extensive period of training is necessary, but for the current task, even this moderate amount of training yielded an advantage for the parent group tested.

Further to this, as might be expected, the longer the duration of music training reported, the bigger the performance advantage on the task. This music advantage in emotion discrimination, especially for those with extensive training, is consistent with previous work showing musician advantages for the recognition of emotion in non-music stimuli such as speech prosody (Lima and Castro, 2011), and pitch processing in speech (Schön et al., 2004; Marques et al., 2007).

The advantage found here for parents with music training is in line with descriptions of the inherent musical nature of mother-infant interactions. Infants are especially responsive to the richly intoned sounds of infant-directed speech (often called motherese), preferring them to the more muted tones of adult-directed speech (Soderstrom, 2007). Mothers across cultures, and indeed across historical periods, sing to their infants, and do so in a distinctive manner marked by high pitch, slow tempo, and emotional expressiveness (Trehub, 2001). This has led some to suggest that the regular, predictive “pulse” of music may act to enhance emotional co-ordination between mother and infant (Nakata and Trehub, 2004).

### EMPATHY

In non-parents, individual differences in self-reported empathy were associated with sensitivity to infant distress. It might be that before parenthood, baseline individual differences in empathy are important in responding to infant distress. There is some emerging evidence that this is particularly the case for men responding to infant facial expressions (Parsons et al., under review). The current findings are also broadly consistent with work showing that individual differences in empathy moderate responses to crying adult faces (Lockwood et al., 2013). The lack of an effect of empathy for the parent group raises interesting questions. One possibility is that parenthood might act to heighten adults’ empathic response to infants (for review, see Mascaro et al., 2014) rendering individual differences in empathy less critical.

### LIMITATIONS

This study was cross-sectional, and the ideal design for investigating a variable like parenthood would be longitudinal. It would therefore be of interest to follow-up adults with and without music training, and varying in dispositional empathy, as they transition into parenthood. Furthermore, it remains to be shown as to whether differences in the discrimination of infant distress might translate into observable differences in caregiving behavior (for discussion, see Parsons et al., 2014a). Two studies have reported that adults wait longer to respond to infant cries that they had previously rated as sounding less distressed (Wood and Gustafson, 2001) and less aversive (Del Vecchio et al., 2009). One recent study has also shown that adults are more likely to indicate immediate and affectionate caregiving responses to high-pitched cries (Out et al., 2010). Clearly, additional work linking actual, observed parent-infant interactions with perceptions of infant expressions would be important. This would complement some of the recent innovative work examining hormonal changes associated with parenthood in both men and women (e.g., Feldman et al., 2013; Mascaro et al., 2014; Weisman et al., 2014).

We included just one question on participants’ formal music training. Our current findings provide impetus for exploring the association between music experience and auditory emotion processing advantages. For instance, it might be that music experience, without formal training, may be sufficient for some individuals to show advantages. There may also be differences dependent on the type of instrument (voice, string, etc.), whether music practice is ongoing, or the age of onset of training. Furthermore, it is unclear why musical effects were apparent only in the parent group and not the non-parent group. Identification of the specifics of music training that confer measurable advantages on emotion processing tasks would be of great interest.

### FURTHER DIRECTIONS

The finding of a music advantage for parents suggests that it might be possible to train adults to detect acoustic parameters communicating infant emotion. Pitch has been robustly demonstrated as an important indicator of distress in this study and in a body of other work (e.g., Dessureau et al., 1998; Schuetze and Zeskind, 2001; Soltis, 2004). A number of studies have shown that it is possible to improve individuals’ performance on pitch discrimination tasks following 30-min training sessions (Amitay et al., 2006a,b). Whether this improvement can generalize to other settings and sounds is currently being debated (Wright and Sabin, 2007).

Furthermore, being able to discriminate emotion accurately does not mean that sensitive, responsive behavior will ensue. However, of potential relevance here are the promising findings from studies of attention or cognitive bias modification training. One study found that training to improve facial emotion recognition can translate into therapeutically relevant effects that persist for at least two weeks (Penton-Voak et al., 2012). Larger scale studies with clinical populations are ongoing (e.g., Adams et al., 2013), but may provide further impetus for implementing emotion discrimination training. If such training is effective, it would be beneficial to parenting contexts because it could be used during pregnancy, and delivered automatically via computer, Internet or smartphone. Such training on emotion discrimination might be helpful in augmenting other early parenting interventions, which have good evidence bases, such as Video Feedback (e.g., Stein et al., 2006; Bakermans-Kranenburg et al., 2008; Lawrence et al., 2013).

### Conflict of Interest Statement

The authors declare that the research was conducted in the absence of any commercial or financial relationships that could be construed as a potential conflict of interest.
